# Supplementary material for: The Construction and Exploration of a Comprehensive MicroRNA Centered Regulatory Network in Foxtail Millet (Setaria italica L.)
Source: Front Plant Sci. 2022 May 6;13:848474. doi: 10.3389/fpls.2022.848474 (PMC9121102; doi:10.3389/fpls.2022.848474)
Supplement: Supplementary file 1 [file Data_Sheet_1.zip › Sit-miR396a_Seita.4G012400.1_809_TPlot.pdf]

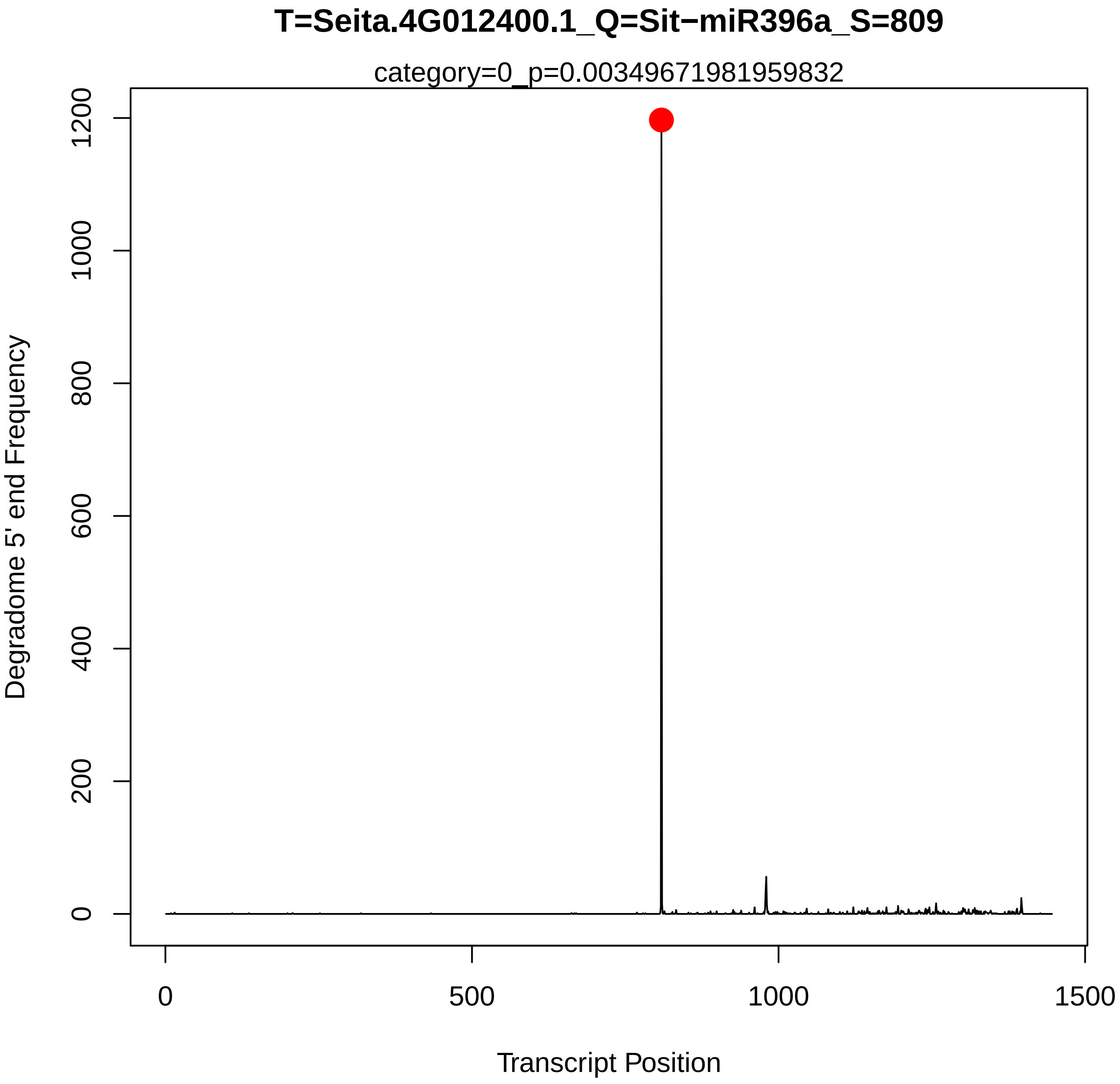

T=Seita.4G012400.1_Q=Sit−miR396a_S=809
category=0_p=0.00349671981959832
Degradome 5' end Frequency
0
200
400
600
800
1000
1200
0
500
1000
1500
Transcript Position
